# THE ASSOCIATION BETWEEN SOCIAL DETERMINANTS OF HEALTH AND SOCIAL FUNCTIONING AMONG OLDER ADULTS WITH STROKE

**DOI:** 10.1093/geroni/igae098.3735

**Published:** 2024-12-31

**Authors:** Lalipat Phianhasin, Chitchanok Benjasirisan, Suebsarn Ruksakulpiwat, Eeeseung Byun

**Affiliations:** University of Washington, Seattle, Washington, United States; Johns Hopkins University, Baltimore, Maryland, United States; Faculty of Nursing, Mahidol University, Nakhon Pathom, Nakhon Pathom, Thailand; University of Washington School of Nursing, Seattle, Washington, United States

## Abstract

Older adults with stroke often experience physical and mental disabilities, resulting in limitations on social functioning, such as participating in social roles and activities. Social determinants of health (SDOH), such as education and income, are shown to have an impact on morbidity and mortality in stroke survivors. However, little is known about the impact of SDOH on social functioning in older adults with stroke. We aimed to examine the associations between SDOH (i.e., marital status, race/ethnicity, education, poverty income ratio [PIR], work status, and health insurance status) and social functioning (i.e., difficulty doing errands alone, participating in social activities, and work limitations due to health problems) in older adults with stroke. We used the National Health Interview Survey 2019-2022 data, which had 61,957 participants aged 60 years and older, including 4,163 stroke survivors. We used logistic regression to examine the associations between SDOH and social functioning after adjusting for covariates, including age, sex, and perceived health status. Being a racial/ethnic minority and not working over the previous week were associated with difficulty doing errands alone. Not being married, being Hispanic or non-Hispanic Asian, and not working over the previous week were associated with difficulty participating in social activities. Not being married, having a PIR < 1 (income below the federal poverty threshold), and not working over the previous week were associated with work limitations due to health problems. Addressing SDOH in care plans for older adults with stroke may improve social functioning and quality of life in this at-risk population.

